# [FeFe] Hydrogenase: Protonation of {2Fe3S} Systems and Formation of Super-reduced Hydride States**

**DOI:** 10.1002/anie.201406210

**Published:** 2014-07-30

**Authors:** Aušra Jablonskytė, Joseph A Wright, Shirley A Fairhurst, Lee R Webster, Christopher J Pickett

**Affiliations:** Energy Materials Laboratory, School of Chemistry, University of East AngliaNorwich Research Park, Norwich NR4 7TJ (UK); John Innes Centre, Norwich Research ParkNorwich NR4 7UH (UK)

**Keywords:** density functional calculations, electrochemistry, enzyme models, hydrides, iron

## Abstract

The synthesis and crystallographic characterization of a complex possessing a well-defined {2Fe3S(μ-H)} core gives access to a paramagnetic bridging hydride with retention of the core geometry. Chemistry of this 35-electron species within the confines of a thin-layer FTIR spectro-electrochemistry cell provides evidence for a unprecedented super-reduced Fe^I^(μ-H)Fe^I^ intermediate.

Hydrides at metallo-sulfur centers play a crucial role in key biological catalysis. These roles include nitrogen fixation,[1] hydrogen evolution/uptake by the [FeFe] and [NiFe] hydrogenases,[2] heterolytic cleavage of molecular hydrogen at [Fe] hydrogenase,[3,4] and such hydrides are likely involved in enzymic catalysis of the interconversion of CO_2_ and CO.[5] They have an implicit role as intermediates in electrocatalytic bioinorganic systems which model natural processes.[6]

Synthetic bridging hydride species at {2Fe2S} cores are structurally well-established[7–9] and the chemistry of terminal hydrides at such centers is rapidly developing.[10–13] Related {2Fe3S} cores have a somewhat closer structural homology to the iron–sulfur core of the [FeFe]-hydrogenase subsite. They have provided primary evidence for bridging carbonyl intermediates at Fe^I^Fe^I^ [14] and Fe^I^Fe^II^ [15] levels and pathways for the synthesis of free-standing[16] or polymer-confined[17] thiolate-bridged {4Fe4S}-{2Fe3S} assemblies.

Herein we describe the synthesis and crystallographic characterization of a complex possessing an unprecedented {2Fe3S(μ-H)} core. We show that this diamagnetic Fe^II^Fe^II^ species undergoes one-electron reduction to a paramagnetic Fe^II^Fe^I^ state with conservation of the {2Fe3S(μ-H)} geometry.[18] Within the confines of a thin-layer spectro-electrochemical cell, further chemistry of this 35-electron species is revealed, and is uncomplicated by reactions with the parent acid complex. Thus decoordination of the thioether ligand and an additional electron transfer is shown to generate an unprecedented super-reduced {Fe^I^(μ-H)Fe^I^} level which possesses an open coordination site on an iron center.

Reaction of the known {2Fe3S}-pentacarbonyl **1**[14] with PMe_3_ gave the complex **2**, which was readily converted into [**3**][PF_6_] by protonation (Scheme 1). The solid-state structures of **2** and the {2Fe3S(μ-H)} complex [**3**][PF_6_] were confirmed by single-crystal X-ray crystallography (see the Supporting Information and Figure 1, respectively). Spectroscopic and analytical data fully supported the formulation of the bulk material (see the Supporting Information).

**Figure 1:**
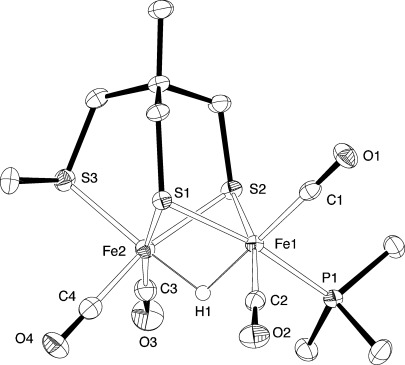
ORTEP representation of the cation of [3][PF_6_] showing 50 % probability ellipsoids. The counter-ion (PF_6_^−^) and hydrogen atoms except H1 have been omitted for clarity.[28]

**Scheme 1:**
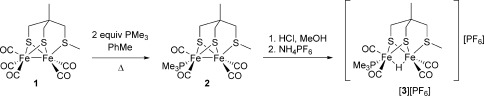
Synthesis of the protonated {2Fe3S} complex.

Cyclic voltammetry (CV) at vitreous carbon shows that 3^+^ is reduced reversibly in a one-electron diffusion-controlled process [*E*_1/2_=−1.12 V versus ferrocenium/ferrocene (Fc^+^/Fc); 0.1 m [NBu_4_][BF_4_]-MeCN; 100 mV s^−1^]. This reduction parallels the behavior of [HFe_2_(pdt)(CO)_4_(PMe_3_)_2_]^+^, except the half-life of the 35-electron {2Fe3S} complex is about five times that of the corresponding {2Fe2S} species.[18] Thin-layer FTIR spectro-electrochemistry shows that upon changing the potential to −1.45 V the signals for 3^+^ at 2006, 2038, and 2061 cm^−1^ are rapidly lost and replaced by a new set of bands at 1992, 1967, and 1915 cm^−1^ (Figure 2, blue spectrum). Density functional theory (DFT) simulation supports the formulation of **3** as the mixed-valence 35-electron species in which no ligand rearrangement has occurred (Scheme 2; see the Supporting Information for calculated spectra).

**Figure 2:**
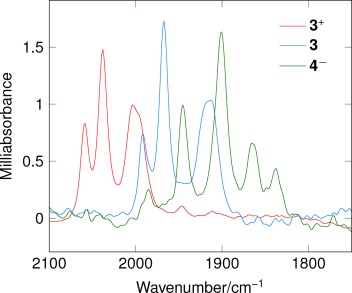
FTIR spectro-electrochemistry spectra for reduction of [3][PF_6_] ([3^+^]_0_ 10 mm in 0.1 m [Bu_4_N][BF_4_]-MeCN, layer thickness 10 μm, reduction potential −1.54 V vs. Fc^+^/Fc). Spectra for 3 and 4^−^ were obtained at 1.2 s and 5.0 s, respectively, after the potential step.

**Scheme 2:**
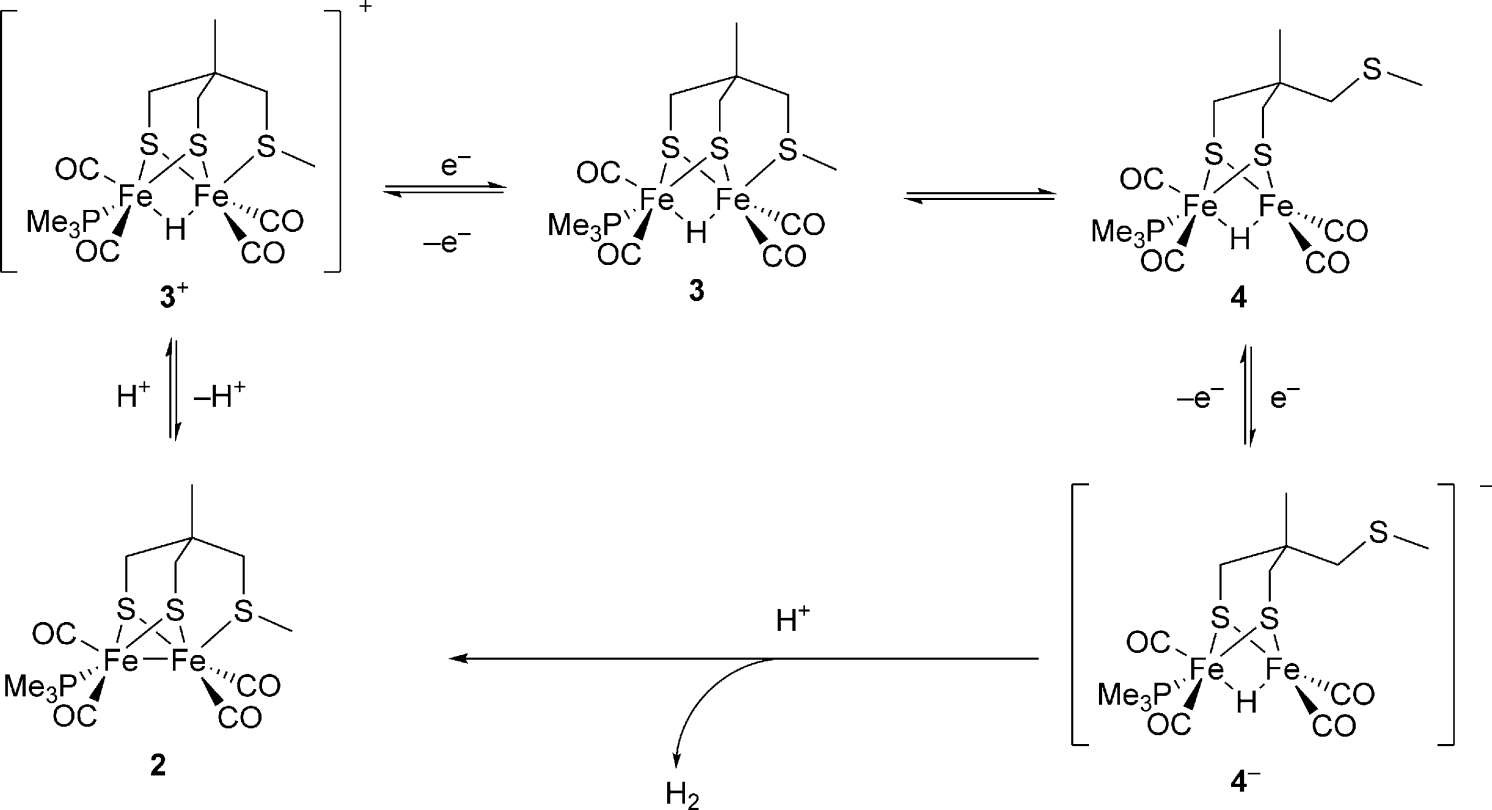
Formation and behavior of the super-reduced state.

Chemical reduction of **3**^+^ using the acenaphthylene monoanion radical allowed examination of **3** by electron paramagnetic resonance (EPR) spectroscopy (see the Supporting Information). At 165 K in THF, a well-defined isotropic spectrum for the *S*=1/2 species is observed. Analysis of the spectrum gives a *g* factor of 2.0209 with strong coupling to the bridging hydride (*A*_iso_=74 MHz) and to the phosphorus atom (*A*_iso_=41 MHz; see the Supporting Information for simulated spectra). The assignment of the two couplings was confirmed using [D]-**3**, which exhibits the expected change in coupling pattern. The absolute coupling values are very similar to those in the symmetrical species [HFe_2_(pdt)(CO)_4_(PMe_3_)_2_][18] and the EPR data are therefore fully in accord with the formulation of **3** as a mixed-valence 35-electron species.

The rapid generation of **3** as a standing solution within the confines of the thin-layer spectro-electrochemical cell provides the means of studying its reactivity without the complication of diffusion into bulk solution where reactions with the parent material can take place. Thus in the thin-layer spectro-electrochemistry experiments on **3**^+^, switching to an open circuit after 2 seconds gives a spectrum for **3** and it is stable for about 3 seconds (see the Supporting Information). However, when the reduction potential is maintained at −1.54 V vs. Fc^+^/Fc the signals for **3** are lost and a new set of peaks grow in concertedly at 1839, 1866, 1900 and 1945 cm^−1^ within a six-second timeframe (Figure 2, green spectrum). We assign these peaks to the production of a single species formed by one-electron reduction of the paramagnetic **3** to the super-reduced bridging hydride **4**^−^_,_ in which the thioether ligand has dissociated (Scheme 2).[19] Strong evidence in support of this assignment is based upon the following observations.

1. Re-oxidation of either the super-reduced {2Fe3S} or {2Fe2S} species in the thin-layer cell regenerates the parent cation. Thus for **3**^+^ reduction at −1.54 V vs. Fc^+^/Fc gives the super-reduced species and application of a potential of −0.75 V vs. Fc^+^/Fc then restores the initial IR bands. This process gives essentially quantitative recovery for **3**^+^, and is substantially greater than that for [HFe_2_(pdt)(CO)_4_(PMe_3_)_2_]^+^. The anchoring of the SMe group to the dithiolate ligand framework explains the high recovery of the parent {2Fe3S} material upon reoxidation. In contrast, for the {2Fe2S} case the chelate effect is absent and PMe_3_ can diffuse from the solvent cage. This diffusion will limit chemical reversibility, as is indeed observed.

2. The spectrum obtained for the thin-layer reduction of [HFe_2_(pdt)(CO)_4_(PMe_3_)_2_]^+^ over a similar time frame is identical to that obtained for the reduction of **3^+^**_,_ with peaks at 1839, 1866, 1900, and 1945 cm^−1^ (see the Supporting Information). This spectrum can only be accommodated if the super-reduced {2Fe3S} and {2Fe2S} species possess identical core frameworks: it must be a consequence of thioether and tertiary phosphine dissociation, respectively.

3. Infrared data were obtained from DFT simulations of the established structure of **3**^+^, its primary one-electron product **3**, and the proposed structure for **4**^−^: Figure 3 shows a good correlation between the experimental and simulated data across all three complexes (*R*^2^=0.983). Spectro-electrochemistry of the deuterated species [DFe_2_(pdt)(CO)_4_(PMe_3_)_2_]^+^ excludes the formation of terminal hydrides as the experimental FTIR spectrum is essentially identical (±2 cm^−1^) to that of the protio species, whereas DFT calculations clearly predict distinct spectra.

**Figure 3:**
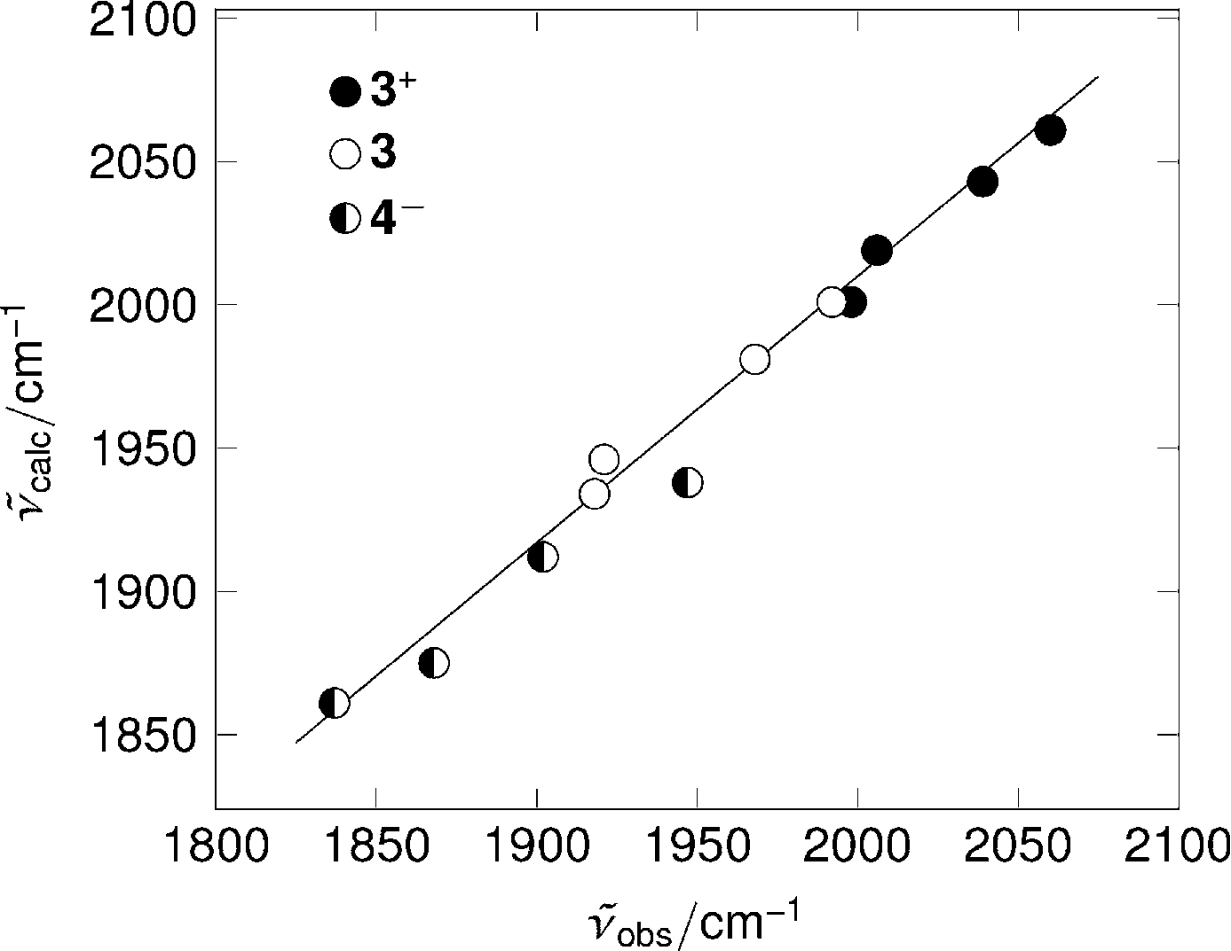
Comparison of observed and calculated infrared frequencies for 3^+^, 3, and 4^−^. The line shows the least-squares fit for all data points.

4. Computational simulations rule out the involvement of thiolate dissociation or of tetranuclear species.[20–22] The latter are predicted to be high in energy and give calculated IR spectra which are in poor agreement with the experimental data (see the Supporting Information), and attempts to minimize super-reduced {2Fe3S} structures with a dissociated thiolate always resulted in re-formation of the Fe=S bond.

The super-reduced species **4**^−^ cannot be observed on the CV timescale. Slow decoordination of the thioether must precede the second electron transfer, and even at slow scan rates (10 mV s^−1^) the only couple observed was for **3**^+^/**3**. This precludes examination of any electrocatalytic hydrogen by **4**^−^ (which is observed but slow for the **3**^+^/**3** couple in the presence of strong acids).

Chemical reduction under stopped-flow conditions can be used to generate the super-reduced species. Reaction of **3**^+^ with [Cp*_2_Co] (Cp*=pentamethylcyclopentadienyl) in MeCN yields **4**^−^ in a flow cell, but not when the same reaction is carried out in bulk, where only unprotonated **2** is obtained. The instability of **4**^−^ under normal synthetic conditions stems from the reaction of the super-reduced state with unreduced **3**^+^. In the SEC and flow experiments, the restricted reaction volume means that **3**^+^ is rapidly reduced to **3**, thus forming **4**^−^ without sacrificial consumption of the parent acid **3**^+^. In contrast, in bulk electrolysis experiments diffusion of **4**^−^ from the reaction layer into the bulk allows attack by **3**^+^ to yield **2** and dihydrogen, which has been directly detected in closely related systems. Similarly, in preparative experiments where mixing is considerably slower than in the stopped-flow chamber, the same proton-transfer chemistry can occur.

A spectator bridging hydride in a 35-electron system which yields dihydrogen upon protonation/disproportionation[23] together with the formation of a related mixed terminal hydride/bridging hydride have been reported.[24] The latter dihydride, [(μ-H)Fe_2_(pdt)(dppv)_2_(CO)(t-H)] [pdt=propane-1,3-dithiolate, dppv=1,2-bis(diphenylphosphino)ethylene], has the terminal hydride located in the basal position: the monoanionic μ-H reported in this work can be viewed as an analogue of the conjugate base of this dihydride.

In summary, synthesis of the first protonated {2Fe3S} core has allowed examination of the reductive chemistry of these biologically relevant species. Single-electron transfer leads to a [Fe^I^(μ-H)Fe^II^] paramagnetic state, which after slow dissociation of the thioether ligand undergoes a second electron transfer to give an unprecedented super-reduced [Fe^I^(μ-H)Fe^I^] state. In the enzyme, the H_sred_ (super-reduced) state is implicated in catalysis,[25] and while **4**^−^ is not a close model for H_sred_ it is notable that we are able to confirm the accessibility of discrete Fe^I^Fe^I^ hydrides.

In a general context, we may note that ligand dissociation or rearrangement following electron transfer to a closed-shell system at which an additional proton can bind is implicated in all electrocatalytic systems based on diiron dithiolate cores. Examples of this are CO and/or thiolate dissociation from hexacarbonyl electrocatalysts[26] and the switch from terminal to bridging CO in certain tetranuclear systems.[27]

## Experimental Section

A solution of **1** (0.70 g, 1.7 mmol) in toluene (100 mL) was treated with PMe_3_ (0.35 mL, 3.4 mmol). The dark red reaction mixture was refluxed under N_2_ atmosphere for 2 h. The solution was then filtered through Celite and the solvent removed in vacuo leaving dark red residue. It was purified by silica chromatography eluting with *n*-hexane/diethyl ether (1:1) mixture. The product was collected as second dark brown fraction. Removal of solvent gave a dark red-brown powder (0.3 g, 32 %) which could be recrystallized from diethyl ether to give crystals of **2** suitable for X-ray diffraction. C,H analysis (%) found (calcd) for C_13_H_21_Fe_2_O_4_PS_3_: C 32.85 (32.52), H 4.29 (4.41); $\tilde \nu $
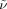
_max_ (MeCN) 1910, 1947, 1984 cm^−1^; ^31^P NMR (121 MHz, CD_3_CN): *δ*=19.98 ppm; *m*/*z* (EI+) 480 (*M*^+^), 452 ([*M*−CO−PMe_3_]^+^), 424 ([*M*−2 CO−PMe_3_]^+^), 396 ([*M*−3CO−PMe_3_]^+^), 368 ([*M*−4 CO−PMe_3_]^+^). Variable-temperature ^31^P NMR spectroscopy in [D_6_]acetone showed that at low temperature (−80 °C) the single phosphorus signal for PMe_3_ group (26.2 ppm) is resolved into two peaks of approximately equal intensity at 29.4 ppm and 29.6 ppm, and is consistent with interconversion of the cisoid and transoid isomers in solution.

A solution of **2** (62.5 mg, 0.13 mmol) in methanol (10 mL) was treated with conc. hydrochloric acid (10 mL) and left to stir under N_2_ atmosphere for 1 hour. A red solid precipitated upon addition of saturated aqueous solution of NH_4_PF_6_ to the solution. The solid was filtered, washed with water and diethyl ether to give product as dark red powder (58 mg, 71 %). C,H analysis (%) found (calcd) for C_13_H_22_Fe_2_O_4_P_2_S_3_: C 24.80 (24.93), H 3.65 (3.54); $\tilde \nu $
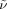
_max_ (MeCN) 2003, 2038, 2060 cm^−1^; ^1^H NMR (300 MHz, CD_3_CN): *δ*=−19.95 ppm (d, 1 H, *J*=21 Hz, hydride); ^31^P NMR (121 MHz, CD_3_CN): *δ*=19.75, 20.21 (PMe_3_, cisoid and transoid isomers), −144.64 ppm (p, *J*=706.5 Hz, PF_6_); *m*/*z* (EI+) 480 ([*M*−PF_6_−H]^+^), 452 ([*M*−CO−PF_6_−H]^+^.

## References

[1] Hoffman BM, Lukoyanov D, Dean DR, Seefeldt LC (2013). Acc. Chem. Res.

[2] Tard C, Pickett CJ (2009). Chem. Rev.

[3] Wright JA, Turrell PJ, Pickett CJ (2010). Organometallics.

[4] Shima S, Ermler U (2011). Eur. J. Inorg. Chem.

[5] Amara P, Mouesca J-M, Volbeda A, Fontecilla-Camps JC (2011). Inorg. Chem.

[6] Gloaguen F, Rauchfuss TB (2009). Chem. Soc. Rev.

[7] Zhao X, Georgakaki IP, Miller ML, Yarbrough JC, Darensbourg MY (2001). J. Am. Chem. Soc.

[8] Zhao X, Georgakaki IP, Miller ML, Mejia-Rodriguez R, Chiang C-Y, Darensbourg MY (2002). Inorg. Chem.

[9] Gloaguen F, Lawrence JD, Rauchfuss TB, Bénard M, Rohmer M-M (2002). Inorg. Chem.

[10] Van der Vlugt JI, Rauchfuss TB, Whaley CM, Wilson SR (2005). J. Am. Chem. Soc.

[11] Ezzaher S, Capon J-F, Gloaguen F, Pétillon FY, Schollhammer P, Talarmin J (2007). Inorg. Chem.

[12] Barton BE, Rauchfuss TB (2008). Inorg. Chem.

[13] Zaffaroni R, Rauchfuss TB, Gray DL, De Gioia L, Zampella G (2012). J. Am. Chem. Soc.

[14] Razavet M, Davies SC, Hughes DL, Pickett CJ (2001). Chem. Commun.

[15] Razavet M, Borg SJ, George SJ, Best SP, Fairhurst SA, Pickett CJ (2002). Chem. Commun.

[16] Tard C, Liu X, Ibrahim SK, De Gioia L, Davies SC, Yang X, Wang L-S, Sawers G, Pickett CJ (2005). Nature.

[17] Ibrahim S, Woi PM, Alias Y, Pickett CJ (2010). Chem. Commun.

[18] Jablonskytė A, Wright JA, Fairhurst SA;, Peck JNT, Ibrahim SK, Oganesyan VS, Pickett CJ (2011). J. Am. Chem. Soc.

[19] Pickett CJ, Dissociation of a single phosphorus atom in bidentate phosphine complexes has been observed following electron transfer: (1984). Electrochemistry, Vol. 4.

[20] Ott S, Kritikos M, Åkermark B, Sun L, Lomoth R Angew. Chem.

[21] Borg S, Behrsing T, Best SP, Razavet M, Liu X, Pickett CJ (2004). J. Am. Chem. Soc.

[22] Best SP, Borg S, White JM, Razavet M;, Pickett CJ Chem. Commun.

[23] Wang W, Nilges MJ, Rauchfuss TB, Stein M (2013). J. Am. Chem. Soc.

[24] Wang W, Rauchfuss TB, Zhu L, Zampella G (2014). J. Am. Chem. Soc.

[25] Adamska A, Silakov A, Lambertz C, Rüdiger O, Happe T, Reijerse E, Lubitz W Angew. Chem.

[26] Razavet M, Borg SJ, George SJ, Best SP, Fairhurst SA, Pickett CJ (2002). Chem. Commun.

[27] Cheah MH, Tard C, Borg SJ, Liu X, Ibrahim SK, Pickett CJ, Best SP (2007). J. Am. Chem. Soc.

[28] CCDC 1008132 and 1008133 contains the supplementary crystallographic data for this paper. These data can be obtained free of charge from The Cambridge Crystallographic Data Centre via http://www.ccdc.cam.ac.uk/data_request/cif.

