# Supplementary material for: [FeFe] Hydrogenase: Protonation of {2Fe3S} Systems and Formation of Super-reduced Hydride States
Source: Angew Chem Int Ed Engl. 2014 Jul 30;53(38):10143–6. doi: 10.1002/anie.201406210 (PMC4497599; doi:10.1002/anie.201406210)
Supplement: Supplementary file 1 [file anie0053-10143-sd1.pdf]

Angewandte Chemie
Eine Zeitschrift der Gesellschaft Deutscher Chemiker

Supporting Information

© Wiley-VCH 2014

69451 Weinheim, Germany

[FeFe] Hydrogenase: Protonation of {2Fe3S} Systems and Formation of Super-reduced Hydride States**

*Aušra Jablonskytė, Joseph A. Wright, Shirley A. Fairhurst, Lee R. Webster, and Christopher J. Pickett**

anie_201406210_sm_miscellaneous_information.pdf

Contents

1 Experimental **2**

1.1 General . . . 2

1.2 [Fe_2{$MeSCH_2C(Me)(CH_2S)_2$}$(CO)_4(PMe_3)$] (**2**) . . . 2

1.3 [HFe_2{$MeSCH_2C(Me)(CH_2S)_2$}$(CO)_4(PMe_3)$][PF_6] ([**3**][PF_6]) . . . 2

1.4 X-Ray crystallography . . . 3

1.5 Electrochemical measurements . . . 4

1.6 Spectrolectrochemical measurements . . . 5

1.7 EPR measurements . . . 7

2 Density functional theory (DFT) calculations **9**

2.1 Atomic coordinates for $\mathbf{3}^+$ . . . 11

2.2 Atomic coordinates for **3** . . . 12

2.3 Atomic coordinates for $\mathbf{4}^-$ . . . 13

2.4 Atomic coordinates for (−"H)$Fe_2(pdt)(CO)_4(PMe_3)^-$ . . . 13

2.5 Atomic coordinates for (T-H)$Fe_2(pdt)(CO)_4(PMe_3)^-$ . . . 14

2.6 Atomic coordinates for [(μ-H)$Fe_2(pdt)(CO)_3(PMe_3)$(μ-CO)]$_2^{2-}$ . . . 15

1 Experimental

1.1 General

Unless otherwise stated, reactions were carried out under nitrogen using conventional air-sensitive techniques. Solvents were degassed using a nitrogen purge and dried using an M. Braun solvent purifier unit before use. Starting materials were purchased from Aldrich or Alfa Aesar and were used without further purification. NMR spectra were recorded on a Bruker Advance 300 system; infra-red spectra were acquired using a Bruker Vertex 80 spectrometer and Specac Omnicell fitted with CaF_2 windows (path length 50 μm).

Compound **1** was prepared following the literature procedure.[1]

1.2 $[Fe_2\{MeSCH_2C(Me)(CH_2S)_2\}(CO)_4(PMe_3)]$ (2)

A solution of **1** (0.7 g, 1.7 mmol) in toluene (100 mL) was treated with PMe_3 (0.35 mL, 3.4 mmol). The dark red reaction mixture was refluxed under N_2 atmosphere for 2 hours. The solution was then filtered through Celite and the solvent removed in vacuo leaving dark red residue. It was purified by silica chromatography eluting with hexane/diethyl ether (1 : 1) mixture. The product was collected as second dark brown fraction. Removal of solvent gave a dark red-brown powder (0.3 g, 32 %) that could be recrystallized from diethyl ether to give crystals of **2** suitable for X-ray diffraction. Anal. found (calcd) for $C_{13}H_{21}Fe_2O_4PS_3$: C, 32.85 (32.52); H, 4.29 (4.41); ^{31}P NMR (121 MHz, CD_3CN): δ 19.98 ppm; m/z (EI+) 480 (M^+), 452 ($[M - CO - PMe_3]^+$), 424 ($[M - 2CO - PMe_3]^+$), 396 ($[M - 3CO - PMe_3]^+$), 368 ($[M - 4CO - PMe_3]^+$); $\tilde{\nu}_{max}/cm^{-1}$ (MeCN) 1910, 1947, 1984.

Variable temperature ^{31}P NMR spectroscopy in d_6-acetone showed that at low temperature (−80 °C) the single phosphorus signal for PMe_3 group (26.2 ppm) is resolved into two peaks of approximately equal intensity at 29.4 ppm and 29.6 ppm, consistent with interconversion of the cisoid and transoid isomers in solution.

1.3 $[HFe_2\{MeSCH_2C(Me)(CH_2S)_2\}(CO)_4(PMe_3)][PF_6]$ ([3][PF$_6$])

A solution of **2** (62.5 mg, 0.13 mmol) in methanol (10 mL) was treated with conc. hydrochloric acid (10 mL) and left to stir under N_2 atmosphere for 1 hour. A red solid precipitated upon addition of saturated aqueous solution of NH_4PF_6 to the solution. The solid was filtered, washed with water and diethyl ether to give product as dark red powder (58 mg, 71 %). Anal. found (calcd) for $C_{13}H_{22}Fe_2O_4P_2S_3$: C, 24.80 (24.93); H, 3.65 (3.54); ^{1}H NMR (300 MHz, CD_3CN) δ −19.95 (d, 1H, J = 21 Hz, Fe−H−Fe); ^{31}P NMR (121 MHz, CD_3CN): δ 19.75, 20.21 (PMe_3, cisoid and transoi isomers), −144.64 (p, J = 706.5 Hz, PF_6); m/z (EI+) 480 ($[M - PF_6 - H]^+$), 452 ($[M - CO - PF_6 - H]^+$. $\tilde{\nu}_{max}/cm^{-1}$ (MeCN) 2003, 2038, 2060.

1.4 X-Ray crystallography

For each sample, crystals were suspended in oil, and one was mounted on a glass fiber and fixed in the cold nitrogen stream of the diffractometer [$T = 140(2)$ K]. Data were collected using Mo-K_α ($\lambda = 0.710\,73$ Å) radiation using an Oxford Diffraction Xcalibur-3 CCD diffractometer equipped with a graphite monochromator and were processed using CrysAlisPro.[2] Structures were determined by the direct methods routines in SIR92 (**3**)[3] or SuperFlip ([**3**][PF_6])[4] and refined by full-matrix least-squares methods on F^2 in SHELXL.[5] Non-hydrogen atoms were refined with anisotropic thermal parameters. The bridging hydrogen atom H(1) in [**3**][PF_6] was located in the Fourier difference map and freely refined; all other hydrogen atoms were included in idealized positions and their U_{iso} values were set toride on the U_{eq} values of the parent atom.

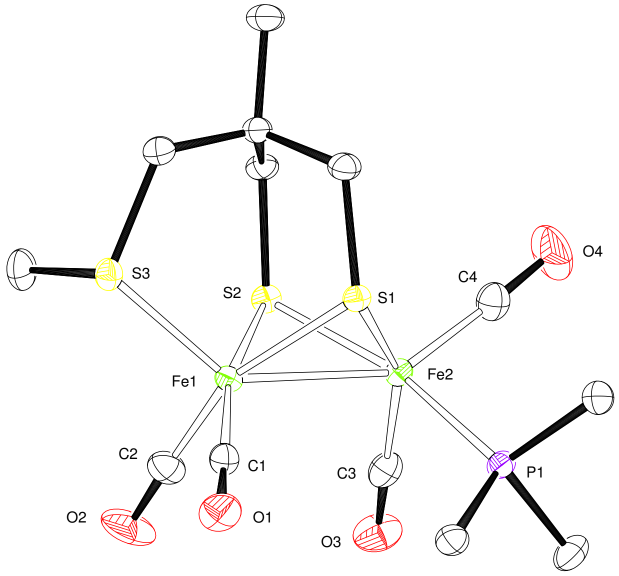

Figure 1: ORTEP representation of the structure of **2** showing 50 % probability ellipsoids; hydrogen atoms have been omitted for clarity.

1.5 Electrochemical measurements

Cyclic voltammetry measurements were carried out in 0.1 M $[NBu_4][BF_4]$-MeCN using a three compartment cell fitted with a glassy carbon working electrode (diameter 3 mm), a platinum counter electrode and a silver wire pseudo-reference electrode interfaced with an Autolab PGSTAT30 potentiostat. Potentials were calibrated using ferrocenium/ferrocene as an internal standard.

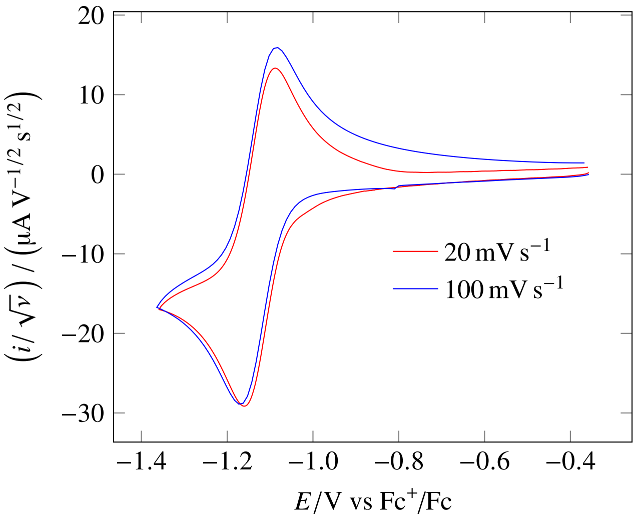

Figure 2: Normalized cyclic voltammograms of [**3**]$[PF_6]$ recorded at a vitreous carbon working electrode in 0.1 M $[Bu_4N][BF_4]$-MeCN at 25 °C.

1.6 Spectrolectrochemical measurements

Infra-red spectroelectrochemical measurements were were carried out in a Spectroelectrochemistry Partners SP-02 cell mounted on a Pike MIRacle ATR fitted with single-bounce silicon top plate. The ATR was fitted with a Northumbria Optical Coatings filter (pass band 4.76 µm to 5.60 µm). Spectra were recorded on a Bruker Vertex 80 spectrometer equipped with a mercury cadmium telluride (MCT) detector. Data were collected at 4 cm^{-1} resolution. Typically, 80 time points were recorded for each experiment. The SP-02 cell was fitted with a glassy carbon working electrode (diameter 3 mm), a platinum counter electrode and a silver wire pseudo-reference electrode. These were attached to a Princeton Applied Research VersaSTAT 3 potentiostat. Data collection was controlled by the Bruker Opus package, interfaced to the potentiostat using a custom Opus3D script and TTL connection. IR data was processed and analysed using Fit_3D (Dr Simon J. George, Lawrence Berkeley National Laboratory, USA) and curve fitting was carried out using SciDAVis.

In a typical run, the cell was purged with nitrogen for some minutes before introduction of the solution for analysis. With the working electrode moved some distance from the ATR crystal (several 100 µm), CV data were acquired to estimate the $E_{1/2}$ value. A series of SEC runs were then carried out, moving the working electrode to within 10 µm of the ATR crystal. After each SEC run, the solution in the cell was allowed to equilibrate with unreacted material.

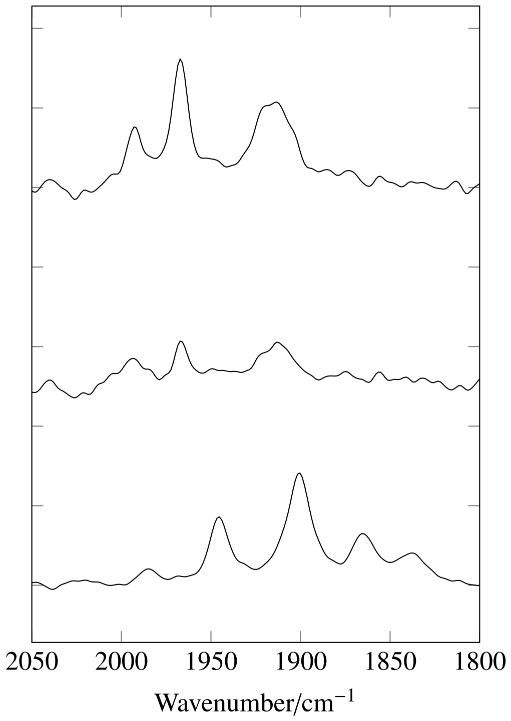

Figure 3: SEC-IR Spectra for reduction of [**3**][PF_6] ([$\mathbf{3}^+$]$_0$ 10 mM in 0.1 M [Bu_4N][BF_4]-MeCN, layer thickness 10 µm, reduction potential −1.2 V vs Ag wire). Top: Reduction for 1 s. Middle: Reduction for 2 s then open circuit for 3 s. Bottom: Reduction for 5 s.

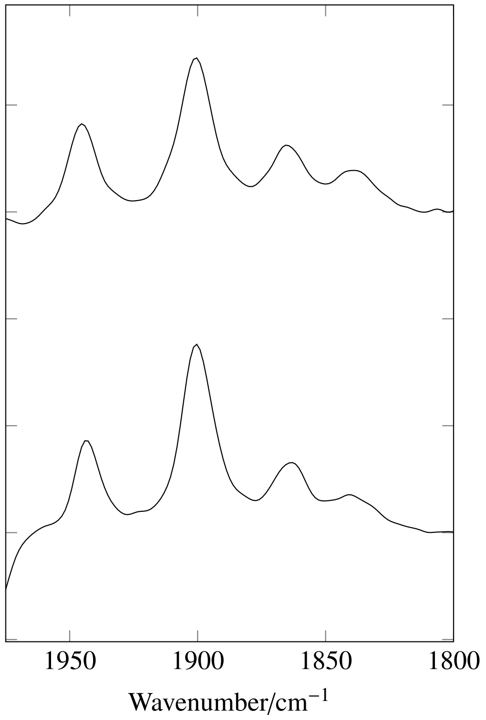

Figure 4: SEC-IR Spectra for two-electron reduction of [**3**][PF_6] (top) and [$HFe_2(pdt)(CO)_4(PMe_3)_2$][$PF_6$] (bottom) in 0.1 M [$Bu_4N$][$BF_4$]-MeCN, layer thickness 10 µm.

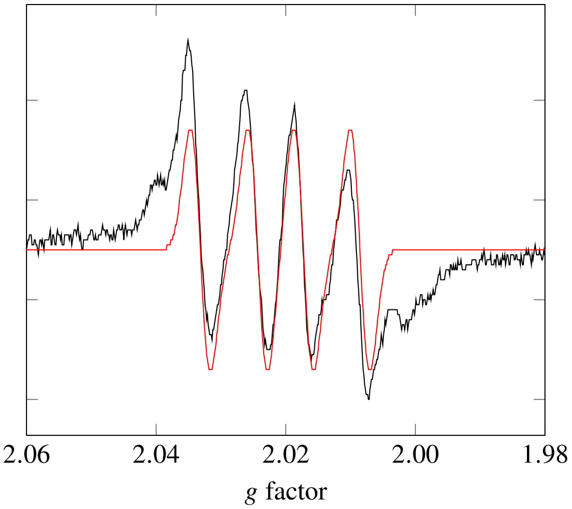

Figure 5: EPR spectra of **3** in THF at 165 K. Black: experimental spectrum; Red: simulated spectrum, g factor = 2.0209, $A_{\text{iso,P}} = -41$ MHz, $A_{\text{iso,H}} = -74$ MHz.

1.7 EPR measurements

A solution of acenaphthalene (6 mg, 0.04 mmol) was prepared in dry THF (10 cm^3). A small amount of finely-divided sodium was added, and the solution was sonicated for approximately one hour, after which time the solution was brown. A second solution of [**3**][PF_6] (7.2 mg, 0.012 mmol) was prepared in dry THF (2 cm^3). Both solutions were then cooled to 195 K. A small portion of the reductant (roughly 0.1 cm^3) was taken up into a syringe, followed by an equal volume of the substrate solution. These were added to a silica EPR tube and rapidly frozen in liquid nitrogen.

Continuous wave EPR spectra were recorded at X-band (9.43 GHz) using a ELEXSYS 500 spectrometer. The samples were warmed to just above the melting point. Temperature control was achieved using a Bruker ER4131VT N2 Temperature Controller. EPR spectra were simulated using the computer programme WINEPR SinFonia (Bruker Analytische Messtechnik GmbH).

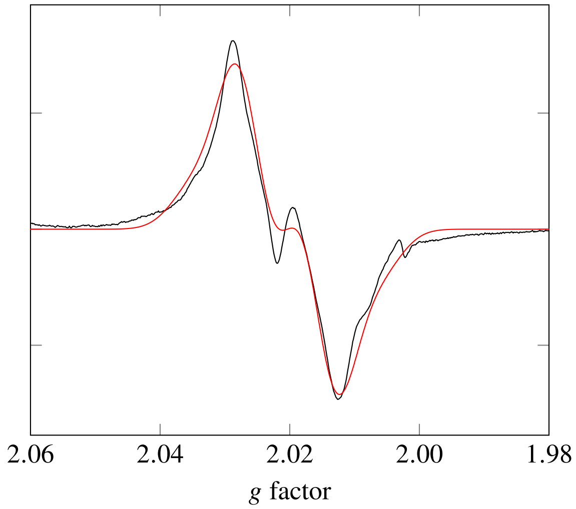

Figure 6: EPR spectra of *deutero*-**3** in THF at 165 K. Black: experimental spectrum; Red: simulated spectrum, g factor = 2.0209, $A_{\mathrm{iso,P}} = -41$ MHz, $A_{\mathrm{iso,D}} = -11.4$ MHz including a 10 % contribution from *protio*-**3**.

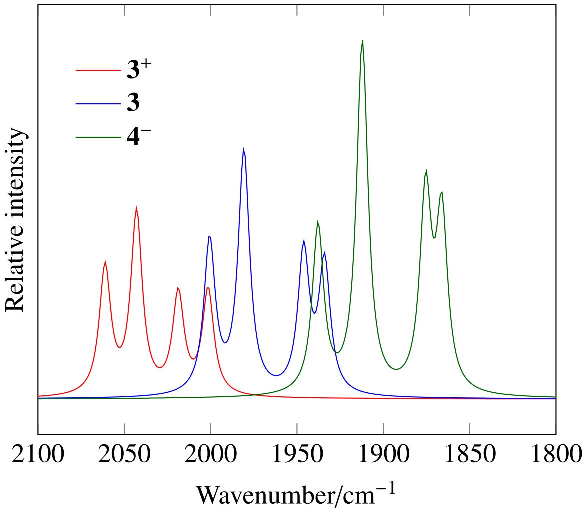

Figure 7: Simulated infrared spectra for structures **3**$^+$, **3** and **4**$^-$.

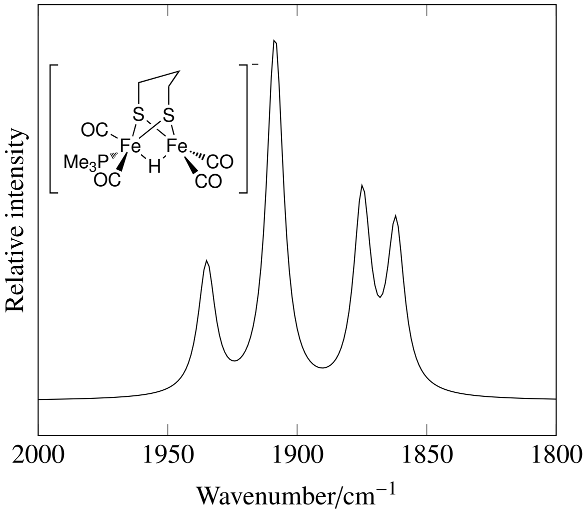

Figure 8: Simulated infrared spectra for (μ-H)Fe_2(pdt)$(CO)_4$$(PMe_3)^-$.

2 Density functional theory (DFT) calculations

All calculations were performed using the Gaussian 09 [6] computational package. Geometry optimisation and frequency calculations have been carried out using the Tao–Perdew–Staroverov–Scuseria [7] (TPSS) density functional. Phosphorus, sulfur and iron atoms are described by the the Hay and Wadt LANL2DZ [8,9] basis set with effective core potential (ECP). In the case of iron, the two outermost p functions were replaced with reoptimized 4p functions. [10] For sulfur and phosphorus, additional p and d polarisation functions were added. [11] All other atoms employ the all electron 6-31+G** basis set. Structures were geometry optimized in the gas phase with the default convergence criteria and confirmed as minima through frequency calculations.

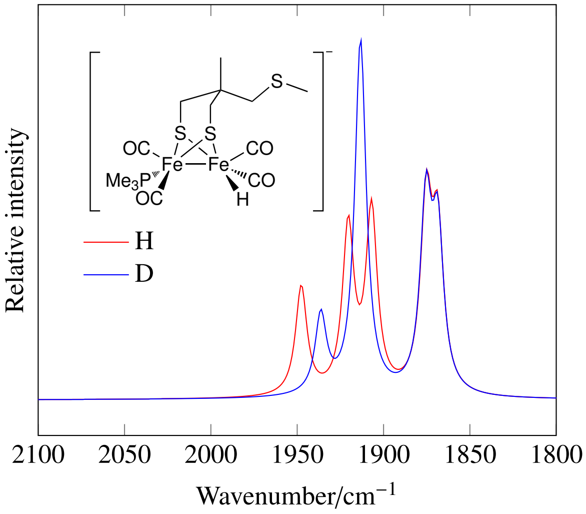

Figure 9: Simulated infrared spectra for reduced terminal hydrides.

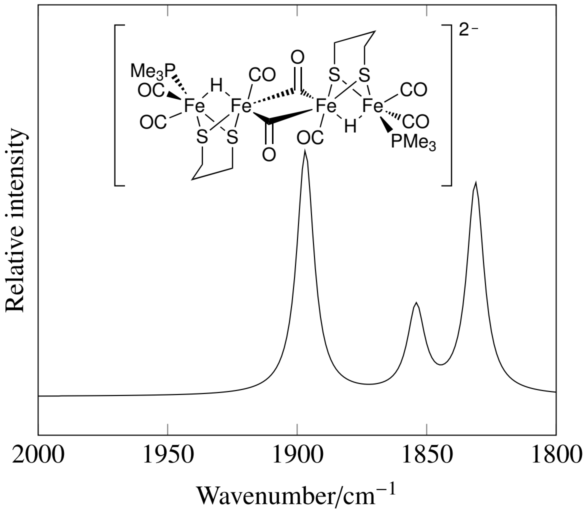

Figure 10: Simulated infrared spectra for $[(\mu\text{-H})Fe_2(pdt)(CO)_3(PMe_3)(\mu\text{-CO})]_2^{2-}$. This structure is 94 $kJ\,mol^{-1}$ per {2Fe2S} unit higher in energy than the equivalent monomeric structure.

2.1 Atomic coordinates for 3^+

C	1.744717268945	0.720565373062	-5.813309911214
C	2.989398571484	-1.912388247615	-5.955985553854
C	4.348651009839	0.269955720593	-4.577815616983
C	-0.894776045085	1.517460288303	-3.549232129157
C	1.270999189553	-2.707038542613	-3.378505924674
C	-1.912706643794	-0.681017824236	-2.673192327849
C	3.197598180547	-1.539156543688	-2.032895119455
C	2.222285937803	1.360623369664	-0.443728620575
C	-2.543088231816	0.405702385823	0.283354320062
C	1.036156828761	-0.650905175644	0.588633185875
C	1.272498885046	0.869053037840	0.663573562719
C	-0.032249833136	1.701349300120	0.712527746539
C	1.991143404953	1.149018733901	2.019009355789
H	2.300010237474	0.912896721825	-6.739350078623
H	3.509796264624	-1.536987074912	-6.845209551670
H	2.040406747435	-2.366930541004	-6.260244730609
H	0.753832774033	0.325400303292	-6.060167895129
H	4.739384821974	0.574361673506	-5.556146367391
H	1.631949897484	1.660431384335	-5.262743668223
H	3.608621959254	-2.675761022403	-5.472414769744
H	5.040837586269	-0.445841963801	-4.121417362011
H	4.262865880453	1.149518094278	-3.930920879154
H	0.238826859829	-0.592333721911	-3.538005501992
H	3.193331059637	0.858835343433	-0.356567353130
H	2.388894150978	2.440132920853	-0.336679104239
H	-3.391002970386	0.259077167034	-0.391307036248
H	-2.090911034720	-0.557492041081	0.533083748982
H	0.217699324147	2.768527445170	0.674409815505
H	1.983924376286	-1.179258702959	0.751535530538
H	-2.879406206533	0.936312732817	1.179593338961
H	0.347459284772	-0.963207442403	1.384672308861
H	-0.561570530206	1.513414294881	1.654356405262
H	2.964769743243	0.644293411310	2.048532573456
H	2.159391282751	2.224729179646	2.157822115002
H	1.391158512837	0.784235398094	2.862423303788
O	-1.196507676297	2.305365703527	-4.345225217503
O	0.992602143719	-3.765967350348	-3.762558978683
O	-2.835389842397	-1.331622843828	-2.939886004915
O	4.188653355258	-1.829822332848	-1.494495508283
P	2.674201765916	-0.509925994493	-4.777597305279
S	1.683308416632	1.148976726484	-2.214531284012
S	0.314644088957	-1.393029379720	-0.960558836086

S	-1.344501998353	1.498856787115	-0.607461453240
Fe	1.676559404593	-1.075466488333	-2.786533121457
Fe	-0.457708202763	0.289119736934	-2.326360099954

2.2 Atomic coordinates for 3

C	1.751479104174	0.662662838177	-5.820000642751
C	3.096791925683	-1.902709880887	-5.915565047337
C	4.330972095919	0.324055385107	-4.531519720623
C	-0.976987069862	1.535961665833	-3.618383070127
C	1.292355724141	-2.722125085524	-3.367441113169
C	-2.023366162430	-0.587593565865	-2.743105457073
C	3.180531055926	-1.520491919362	-1.932615206693
C	2.190637619291	1.347783919330	-0.519275982719
C	-2.513567902639	0.423095990154	0.385278264596
C	0.978324721003	-0.660355076878	0.523249612803
C	1.261584625297	0.855249378926	0.610965926320
C	-0.012360312017	1.730287412527	0.739390834123
C	2.041616268490	1.081976761521	1.941304061317
H	2.328166897296	0.879861350785	-6.728282018079
H	3.614819725121	-1.506329249883	-6.798098883228
H	2.175839963079	-2.405309488539	-6.231011119829
H	0.785415368772	0.223592872761	-6.091028790115
H	4.733686610974	0.624828885928	-5.507045939962
H	1.569712511945	1.593733449451	-5.272585145234
H	3.739899429744	-2.636449210089	-5.416648724654
H	5.034968476473	-0.357101382018	-4.040434160998
H	4.193371840142	1.208308723123	-3.899747587815
H	0.260486136665	-0.648956192757	-3.527479631288
H	3.149052399056	0.815659795291	-0.458482233942
H	2.387907576949	2.419385172903	-0.374479053607
H	-3.423377678349	0.353680767150	-0.218540600954
H	-2.055521617558	-0.568515427033	0.464998359977
H	0.281008909877	2.787495893919	0.705123605277
H	1.921226305383	-1.206559627007	0.662217355049
H	-2.758115208324	0.833138229451	1.371238618912
H	0.309317733814	-0.948260653223	1.346985340844
H	-0.486390460103	1.552283757338	1.713877710784
H	3.001210574248	0.549173855430	1.921609619525
H	2.246410150792	2.150110623900	2.098751694710
H	1.463206132669	0.714570760428	2.800143027728
O	-1.265612866828	2.329140353491	-4.431487130976
O	1.036608396046	-3.801393810077	-3.740873076907
O	-2.979954223455	-1.211651186346	-3.002844053767

O	4.163108661303	-1.841443084398	-1.376419934112
P	2.682382422884	-0.524471832536	-4.728368586064
S	1.610216869574	1.193729738420	-2.280377704866
S	0.197976537507	-1.385485703934	-1.007171041437
S	-1.393846469187	1.588441514148	-0.507046446638
Fe	1.680153283782	-1.104515301143	-2.796131583286
Fe	-0.564586083272	0.335568582007	-2.400164343716

2.3 Atomic coordinates for 4^{-}

C	3.292061	1.264814	1.793539
C	4.867356	-0.295755	-0.057603
C	3.417700	-1.609289	2.056608
C	-0.525715	2.788271	-1.157506
C	2.282499	-0.161356	-2.006314
C	-0.016737	2.657531	1.247864
C	1.390844	-2.276953	-0.639490
C	-1.227309	-1.564341	1.342110
C	-5.998443	-0.459305	0.812012
C	-1.844277	-1.403335	-1.125567
C	-2.380334	-1.394673	0.323040
C	-3.161976	-0.093805	0.642023
C	-3.295271	-2.635872	0.478877
H	1.279624	1.292880	-0.416767
O	-0.711547	3.672721	-1.922964
O	2.892958	0.027610	-2.998123
O	0.131409	3.449982	2.120411
O	1.487722	-3.448691	-0.783843
P	3.179462	-0.283339	0.755555
S	0.083666	-0.240840	1.431215
S	-0.639607	-0.079336	-1.651976
S	-4.716254	0.203948	-0.338389
Fe	1.387063	-0.520802	-0.547050
Fe	-0.230068	1.511031	-0.026516

2.4 Atomic coordinates for $(\mu\text{-H})Fe_2(pdt)(CO)_4(PMe_3)^{-}$

C	0.831217376162	1.861578940701	-2.314440317516
C	-1.506882874283	1.863616442486	0.141645201639
C	2.040533590290	2.437632883883	-1.574382412244
C	2.851382258336	-1.286362145637	1.051383452508
C	-0.966594624432	0.133863025288	2.104199691894
C	1.774214651375	2.757178185768	-0.101553214770
C	1.965048315130	-2.163076100618	-1.071410288372

C	-3.262875123937	-0.491262921862	-1.654282547797
C	-3.438014811190	-1.631022618758	0.988253355116
C	-1.565874955173	-2.715366860354	-0.919744522294
H	-0.047532411598	2.512544505752	-2.205463028296
H	1.060190514462	1.757166777189	-3.384172407756
H	2.875136369860	1.722860174315	-1.641915991106
H	2.349308286860	3.369338101093	-2.084069989308
H	0.429253563172	-1.131978218040	0.616198495992
H	2.650747811774	3.256704719533	0.334822051314
H	-3.828493254029	0.387166902667	-1.321491298125
H	0.907097767973	3.424229821998	0.007010945803
H	-2.620377193591	-0.199096545620	-2.492604639149
H	-3.965505539202	-0.756085979772	1.386250526165
H	-3.958607627993	-1.280212112305	-1.972810937378
H	-0.895604260873	-2.538581486751	-1.767963033052
H	-2.933504537790	-2.135509918285	1.820120965308
H	-0.990846437494	-3.223053502581	-0.138080015712
H	-4.162803908639	-2.317623066282	0.529264699083
H	-2.413850326569	-3.339560674298	-1.235529064829
Fe	1.737535973259	-0.721671310375	-0.146632517243
Fe	-0.602954513290	0.382023567291	0.410820501512
O	-2.171739305100	2.840072923089	0.052979641073
O	3.612588460108	-1.686992867600	1.867185527788
O	-1.243673176704	0.016961471049	3.245515568077
O	2.124383096353	-3.159568485233	-1.698903890576
P	-2.156456657463	-1.071725505761	-0.259842081037
S	0.344409304697	0.144574473473	-1.764504538281
S	1.487856519539	1.271659964559	0.993232451569

2.5 Atomic coordinates for (T-H)Fe_2(pdt)$(CO)_4(PMe_3)^-$

C	-7.757592389482	1.846402795190	-2.985122117487
C	-7.651755478034	-1.000763775501	-2.538634960587
C	-12.232421230141	1.621597713989	-2.076935841042
C	-10.192763702678	-2.408358315019	-0.664388829756
C	-13.227657754515	1.619119953268	-0.915846112956
C	-6.072699846682	0.773559744407	-0.900247222669
C	-9.173412839474	2.597730863137	0.032093481032
C	-12.675935451819	-1.800949272243	-0.153142420545
C	-12.616284998157	2.025226809109	0.426433865285
C	-8.590979569248	0.076228292826	1.130207494853
H	-8.703554268962	1.770978703560	-3.533385817935
H	-6.914875401176	1.657021501791	-3.665027659396
H	-8.592344338162	-1.185668962174	-3.069655275805

H	-6.821355162666	-0.949708302475	-3.257347781432
H	-12.752863650396	1.386120119589	-3.016224648144
H	-7.673990762287	2.854551215801	-2.562963021014
H	-11.754600272667	2.605626345583	-2.182984978061
H	-7.487261655794	-1.823716208448	-1.835691522549
H	-5.317869191669	0.680277420302	-1.693407073135
H	-14.045286116108	2.324326268270	-1.155924100485
H	-13.672584355233	0.618531893136	-0.824935189061
H	-5.976670725516	1.752059100603	-0.415014740547
H	-5.907913434199	-0.003276974273	-0.145005894374
H	-10.818492355172	-1.641702072787	1.226222729516
H	-12.116624031627	3.001082861220	0.355078690298
H	-13.405689456050	2.099985513648	1.187726005602
Fe	-9.474480519676	0.888905877501	-0.150034921168
Fe	-11.118294897523	-1.043616868244	-0.123101748792
O	-9.587329672189	-3.389205518048	-0.930842412149
O	-8.960152387695	3.747260121209	0.239291594810
O	-13.725340354245	-2.349349071760	-0.172771401909
O	-7.939753840555	-0.421909975286	1.980947371228
P	-7.807017118733	0.601592897665	-1.587369556454
S	-10.863263097056	0.354958782846	-1.941691104329
S	-11.375489674412	0.824650521608	1.151835119155

2.6 Atomic coordinates for $[(\mu\text{-H})Fe_2(pdt)(CO)_3(PMe_3)(\mu\text{-CO})]_2^{2-}$

C	-2.654237260739	-2.534944224324	-2.069512579537
C	-5.292750039813	-1.535929173250	0.256633292035
C	-2.880581777960	-3.470151291987	-0.877602789756
C	-0.267868072098	0.857892671353	1.107398778115
C	-4.268736893768	0.102277906951	2.041177627833
C	-1.948904422915	-3.233987358034	0.314405019355
C	-1.202833361889	1.565894882085	-1.253055578286
C	-5.151308843747	1.731167798442	-2.191920666827
C	-6.862245141503	1.513604461135	0.116901648654
C	-4.440721525459	3.066039739605	0.259494596312
H	-3.339227981387	-2.804371443722	-2.886872088993
H	-1.618526071714	-2.602812677820	-2.431031553006
H	-2.721147380300	-4.508675436024	-1.228467312453
H	-3.924653482837	-3.397300702115	-0.544595284555
H	-2.642805692386	0.745734974894	0.350901111263
H	-0.898287262581	-3.321832627998	0.004888917760
H	-5.612122585751	0.867566085889	-2.684592159655
H	-2.141102701449	-3.993458500465	1.085765621349
H	-4.138384440938	1.848960571560	-2.591691228195

H	-7.391104655636	0.643828803892	-0.290975186625
H	-5.741030866018	2.639586737669	-2.385314347365
H	-3.403857030463	3.197274974830	-0.067587118325
H	-6.949204150884	1.485724395630	1.209984058526
H	-4.459725237795	3.067242761898	1.355586962726
H	-7.322665444387	2.437917364844	-0.262326608899
H	-5.062804105210	3.888407896290	-0.125245260028
Fe	-1.261041986982	0.116959731349	-0.307298539562
Fe	-3.951662354360	-0.380224498492	0.398763751035
O	-6.317083803418	-2.138758515394	0.320613126141
O	-0.383421139689	1.586238969286	2.064595105436
O	-4.542401399856	0.403565449220	3.152427198282
O	-1.274760914527	2.577091964889	-1.874058502405
P	-5.046788340346	1.404356510180	-0.352729688346
S	-2.924696152205	-0.720730777930	-1.746909465294
S	-2.082377455445	-1.587471073303	1.182038066824
C	1.948571672764	3.233613468723	-0.314583693227
C	5.292457424629	1.535765180878	-0.257069744322
C	2.880250479795	3.469853364072	0.877410625577
C	0.267696661821	-0.858309875832	-1.107535140420
C	2.653967971647	2.534630117430	2.069317540282
C	1.202655291860	-1.566316115672	1.252856043544
H	2.140690601334	3.993103875852	-1.085944843197
H	0.897955219966	3.321380732713	-0.005038677904
H	2.720753648107	4.508370419764	1.228267033410
H	3.924323076873	3.397063951475	0.544396223006
H	2.642597230731	-0.746134475817	-0.350946936316
H	1.618263590006	2.602461336202	2.430864383042
H	3.338971355676	2.804054069938	2.886666879524
Fe	1.260844214722	-0.117360139445	0.307149081851
Fe	3.951489673593	0.379888533858	-0.398935676992
O	6.316738866767	2.138671065882	-0.321183475257
O	0.383172933665	-1.586721314886	-2.064688063784
O	1.274826808202	-2.577205274950	1.874332802596
S	2.082148695949	1.587126164782	-1.182247049969
S	2.924466281849	0.720431626059	1.746662529336
P	5.046848297842	-1.404421055398	0.352838742643
C	4.268521217119	-0.102760095905	-2.041320104365
C	4.440780066525	-3.066350590714	-0.258727093589
H	4.459319573123	-3.067800841890	-1.354826645844
H	5.063146926358	-3.888537213684	0.125942425655
H	3.404071497431	-3.197676493481	0.068813984039
C	6.862213991322	-1.513692924232	-0.117150185384

H	7.322800276202	-2.437814517354	0.262341619625
H	6.948931364748	-1.486241960156	-1.210262747330
H	7.391087525737	-0.643708688636	0.290264466046
C	5.151824922589	-1.730666906401	2.192111226058
H	5.741633961937	-2.638999369541	2.385645556017
H	5.612724501092	-0.866892760154	2.684402321930
H	4.139003957679	-1.848365435718	2.592171238493
O	4.542475196797	-0.404193208795	-3.152456568308

References

[1] C. Tard, PhD thesis, University of East Anglia, Norwich, UK, **2005**.

[2] Oxford Diffraction Ltd., Abingdon, UK, CrysAlisPro, **2010**.

[3] A. Altomare, G. Cascarano, C. Giacovazzo, A. Guagliardi, *J. Appl. Cryst.* **1993**, *26*, 343–350.

[4] L. Palatinus, G. Chapuis, *J. Appl. Crystallogr.* **2007**, *40*, 786–790.

[5] G. M. Sheldrick, *Acta Cryst. A* **2008**, *64*, 112–122.

[6] M. J. Frisch, G. W. Trucks, H. B. Schlegel, G. E. Scuseria, M. A. Robb, J. R. Cheeseman, G. Scalmani, V. Barone, B. Mennucci, G. A. Petersson, H. Nakatsuji, M. Caricato, X. Li, H. P. Hratchian, A. F. Izmaylov, J. Bloino, G. Zheng, J. L. Sonnenberg, M. Hada, M. Ehara, K. Toyota, R. Fukuda, J. Hasegawa, M. Ishida, T. Nakajima, Y. Honda, O. Kitao, H. Nakai, T. Vreven, J. A. Montgomery, Jr., J. E. Peralta, F. Ogliaro, M. Bearpark, J. J. Heyd, E. Brothers, K. N. Kudin, V. N. Staroverov, R. Kobayashi, J. Normand, K. Raghavachari, A. Rendell, J. C. Burant, S. S. Iyengar, J. Tomasi, M. Cossi, N. Rega, J. M. Millam, M. Klene, J. E. Knox, J. B. Cross, V. Bakken, C. Adamo, J. Jaramillo, R. Gomperts, R. E. Stratmann, O. Yazyev, A. J. Austin, R. Cammi, C. Pomelli, J. W. Ochterski, R. L. Martin, K. Morokuma, V. G. Zakrzewski, G. A. Voth, P. Salvador, J. J. Dannenberg, S. Dapprich, A. D. Daniels, Ö. Farkas, J. B. Foresman, J. V. Ortiz, J. Cioslowski, D. J. Fox, Gaussian 09 Revision C.01, Gaussian, Inc., Wallingford, CT, **2009**.

[7] J. Tao, J. P. Perdew, V. N. Staroverov, E. Scuseria, G., *Phys. Rev. Lett.* **2003**, *91*, 146401–146405.

[8] P. J. Hay, W. R. Wadt, *J. Chem. Phys.* **1985**, *82*, 270–283.

[9] P. J. Hay, W. R. Wadt, *J. Chem. Phys.* **1985**, *82*, 284–298.

[10] M. Couty, M. B. Hall, *J. Comput. Chem.* **1996**, *17*, 1359–1370.

[11] C. E. Check, T. O. Faust, J. M. Bailey, B. J. Wright, T. M. Gilbert, L. S. Sunderlin, *J. Phys. Chem. A* **2001**, *105*, 8111–8116.
